# Uridine Diphosphate Glycosyltransferases (UGTs) Involved in the Carotenoid-Based Body Color Difference between *Tetranychus cinnabarinus* (Red) and *Tetranychus urticae* (Green)

**DOI:** 10.3390/insects14100823

**Published:** 2023-10-18

**Authors:** Zhifeng Xu, Ting Lin, Tongyang Wang, Yuan Hu, Guangmao Shen, Kaiyang Feng, Ping Zhang, Lin He

**Affiliations:** 1 College of Plant Protection, Southwest University, Chongqing 400715, China; 2 Key Laboratory of Agricultural Biosafety and Green Production of Upper Yangtze River (Ministry of Education), Southwest University, Chongqing 400715, China

**Keywords:** carotenoids, body color, *Tetranychus urticae*, Uridine diphosphate glycosyltransferases (UGTs), RNA interference

## Abstract

**Simple Summary:**

There has been ongoing scholarly discourse regarding the potential identity of the carmine spider mite and two-spotted spider mite. However, the most prominent distinction between the two mite species is their body coloration, with the former exhibiting red and the latter displaying green. Our investigation has revealed that the dissimilarity in body pigmentation between the two mite species primarily stems from variations in carotenoid content and composition within their anatomical structures. Furthermore, it is plausible that this dissimilarity may be attributed to the differential expression of genes associated with pigmentation. Following the inhibition of various pigment-related genes, the mites exhibited varying alterations in carotenoid content. Notably, the inhibition of Uridine diphosphate glycosyltransferases induced a transition in body color from green to yellow in the two-spotted spider mite. While the current investigation has not completely elucidated the mechanism underlying the pigmentation disparities between the two mite species, we have conducted an analysis of carotenoid content and the expression of their anabolic genes, thereby establishing a basis for a comprehensive examination of the pigmentation pathway.

**Abstract:**

It has long been disputed whether *Tetranychus cinnabarinus* and *Tetranychus urticae* belong to the same genus, with *T. cinnabarinus* regarded as a red form of *T. urticae*. However, it is unclear why *T. urticae* and *T. cinnabarinus* have different body colors. Since carotenoids are responsible for the color of many organisms, the carotenoid profiles of *T. cinnabarinus* and *T. urticae* were compared by HPLC. There was no difference in carotenoid type, but *T. cinnabarinus* contained significantly more neoxanthin, astaxanthin, α-carotene, β-carotene, and γ-carotene, which may contribute to the deep red color. The transcriptome sequencing of both species identified 4079 differentially expressed genes (DEGs), of which 12 were related to carotenoid metabolism. RNA interference (RNAi) experiments demonstrated that silencing seven of these DEGs resulted in the different accumulation of carotenoid compounds in *T. cinnabarinus* and *T. urticae*. In addition, the body of *T. urticae* turned yellow after two days of feeding with *UGT* double-stranded RNAs and *β-UGT* small interfering RNAs. In conclusion, differences in the carotenoid profiles of *T. urticae* and *T. cinnabarinus* may be responsible for the different body colors.

## 1. Introduction

There has long been controversy over the taxonomy of *Tetranychus urticae* and *Tetranychus cinnabarinus*, with several researchers arguing that the two species are the same (*T. urticae*) [1,2]. The external morphology of both species is very similar, except for the coloration of eggs and adult females, making it difficult to distinguish them morphologically. Insect color differentiation has been linked to numerous factors, including gene expression levels, material transformation and flow distribution, and other synthetic pigments [3,4,5,6]. In genetically similar species, differences in gene expression can result in genotypic variation, e.g., differential expression or the deletion of pigment-related genes can cause phenotypic polymorphisms [7,8]. Carotenoids make up most of the mite pigment composition [9]. Horizontally transferred synthetic genes regulate the synthesis of endogenous carotenoids and are involved in pigmentation and diapause in *T. urticae* [10,11].

Carotenoids are a class of conjugated isoprene molecules widely found in animals, plants, bacteria, fungi, algae, and other organisms, with colors ranging from bright yellow to dark red [9,12]. The first carotenoid synthesized is the colorless phytoene, which then forms phytofluene, ζ-carotene, neurosporene, and lycopene via desaturase activity. Lycopene undergoes different cyclization reactions, including carbonylation, hydroxylation, sugar addiction, and oxidative cleavage, to produce a variety of non-oxygenated carotenoids, such as α-carotene, β-carotene, and γ-carotene, eventually, producing carotenoids such as lutein, neoxanthin, and astaxanthin through oxidase catalysis [9,13,14]. These carotenoids have different colors and functions in living organisms due to their various structures, such as decoration, vision, mechanical protection, stress resistance, signaling, and antioxidants [15,16].

Researchers discovered that horizontal gene transfer enabled carotenoid biosynthesis in pea aphids [17,18]. Additionally, carotenoid biosynthesis genes were discovered in the genomes of *T. urticae*, which can also synthesize carotenoids [19,20]. Different carotenoids are responsible for varying body colors within and between species, and these carotenoid molecules are regulated by related genes. In aphids, the red-green polymorphism is caused by the presence or absence of red carotenoids (torulene), and phytoene desaturase is the key gene that determines whether torulene is synthesized [17]. In *T. urticae*, the carotenoid synthesis genes transferred from the fungal level are involved in diapause and different color morphologies, but the disruption of phytoene desaturase did not affect body color, only resulting in diapause [10,11]. However, mites appear to have a more complex body color formation than aphids.

This study compared the carotenoid profiles of *T. cinnabarinus* and *T. urticae* and used high-throughput sequencing to determine whether the carotenoid-related genes were differentially expressed. RNAi technology was used to validate the function of carotenoid-related differentially expressed genes (DEGs) and establish an understanding of their formation.

## 2. Materials and Methods

### 2.1. Mites

*T. cinnabarinus* and *T. urticae* populations were collected from the same rose plantation in Kunming, Yunnan Province, in May 2014. SS-*T. cinnabarinus* was collected from a cowpea field in Chongqing Province in 1998. *Tetranychus truncatus* was collected from cowpea seedlings in the vegetable garden of Dongguan, Guangdong Province, in May 2019. *Panonychus citri* was gifted by the Innovative Research Team of Insect Molecular Ecology, Institute of Agricultural Sciences, Southwest University. The mite populations were grown on cowpea seedlings using water seals to isolate the populations and avoid cross-contamination in the following conditions: 26 ± 1 °C, relative humidity of 55–75%, and a photoperiod of L 14 h: D 10 h.

### 2.2. Determination of Carotenoid Content

Four hundred adult female 3-day mites were ground and extracted in 200 µL of 66.7% ethanol by sonicating without light for 30 min. The supernatant was concentrated under vacuum for 20 min, dried before adding 1 mL of hexane, and then centrifuged at 10,000 rpm for 30 min. Subsequently, 100 µL of acetonitrile was added to dissolve the pigments, and 40 µL was transferred to a C_30_ carotenoid-specific HPLC column. The chromatographic separation conditions were: column temperature of 25 °C, a flow rate of 1 mL/min, mobile phase A comprising 75% acetonitrile + 25% methanol, and B containing 100% methyl tert-butyl ether (MTBE). The experiments were performed in triplicate. The quantitative analysis of the target substance was carried out according to the content of the standard, and the formula was calculated as follows: $X=\left(\;\mathrm{Sx}/\mathrm{Ss}\cdot C\cdot V/m\right)$. Unit, mg/g; Sx, abundance value of target substance; Ss, abundance value of standard; C, concentration of standard; V, dissolution volume of sample; m, mass of pre-treated sample. The results were analyzed for significant differences using the independent samples *t*-test in SPSS Statistics (22.0) ANOVA software (SPSS, Inc., Chicago, IL, USA).

### 2.3. Transcriptome Sequencing

Total RNA was extracted from two hundred adult female 3-day old mites of *T. cinnabarinus* and *T. urticae* for transcriptome sequencing, as outlined previously [21]. Briefly, transcriptome sequencing was performed using the Illumina HiSeq platform to generate paired-end reads. The raw sequencing data were analyzed using the *T. urticae* genome as a reference for sequence alignment and subsequent analysis. Unigene expression abundance was calculated using fragments per kilobase of transcript per million fragments mapped (FPKM) values, and differentially expressed genes (DEGs) were identified using DESeq2, with Fold Change ≥ 2 and FDR < 0.01 as the criteria.

### 2.4. Fluorescence Quantitative PCR (qPCR)

The DEGs involved in carotenoid metabolism synthesis were screened and validated by qPCR. RNA was reverse transcribed using the TaKaRa PrimeScript^TM^ RT reagent kit. Primers were designed using Primer 3, and *RPS18* (FJ608659) and *α-TUB* (FJ526336) were the internal reference genes (Table S1) [22]. qPCR was performed in 20 µL reaction mixtures using a qTOWER 2.0 (Analytik Jena, Jena, Germany) with the following cycling conditions: 95 °C for 2 min, then 40 cycles of denaturation at 95 °C for 15 s, 60 °C for 30 s, and elongation at 72 °C for 30 s. The relative quantitative method (^ΔΔ^CT) was used to calculate the fold change of target genes [23]. Three biological replicates and two technical duplicates were performed.

### 2.5. RNA Interference (RNAi)

Double-stranded RNAs (dsRNAs) or small interfering RNA (siRNA) were synthesized using the T7 RiboMAX™ Express RNAi System kit according to the manufacturer’s instructions. The RNAi primers are listed in Table S2. RNAi experiments were performed using the leaf disk method, with a dsRNA or siRNA concentration of 1000 ng/μL, a green fluorescent protein (GFP) as a negative control for dsRNA, and water as a negative control for siRNA [24]. The fresh cowpea leaves were cut into approximately 1.5 cm^2^ and subsequently subjected to dehydration in a 60 °C oven. Following dehydration, the leaves were exposed to a dsRNA solution, with 30 μL being added to facilitate complete absorption. Female adult mites were then carefully selected and transferred into 1.5 mL centrifuge tubes, where they were deprived of food for 24 h. Subsequently, Petri dishes, sponges, and water were sterilized at high temperatures. The dehydrated leaves, which had been treated with the dsRNA solution, were placed onto the sponge, and the starved leaf mites were introduced onto the leaves for a duration of 48 h to conduct the RNAi test. Mites were collected for RNAi efficiency assay and carotenoid extraction experiments after 48 h of dsRNA or siRNA feeding. The experiments were performed in triplicate.

### 2.6. Statistical Analysis

The data were analyzed using the Student’s *t*-test or one-way ANOVA followed by Tukey’s test for multiple comparisons using SPSS Statistics 22.0. A *p*-value < 0.05 or 0.01 was considered statistically significant. All results are expressed as the mean ± SEM.

## 3. Results

### 3.1. Carotenoid Profiles

**Figure 1:**
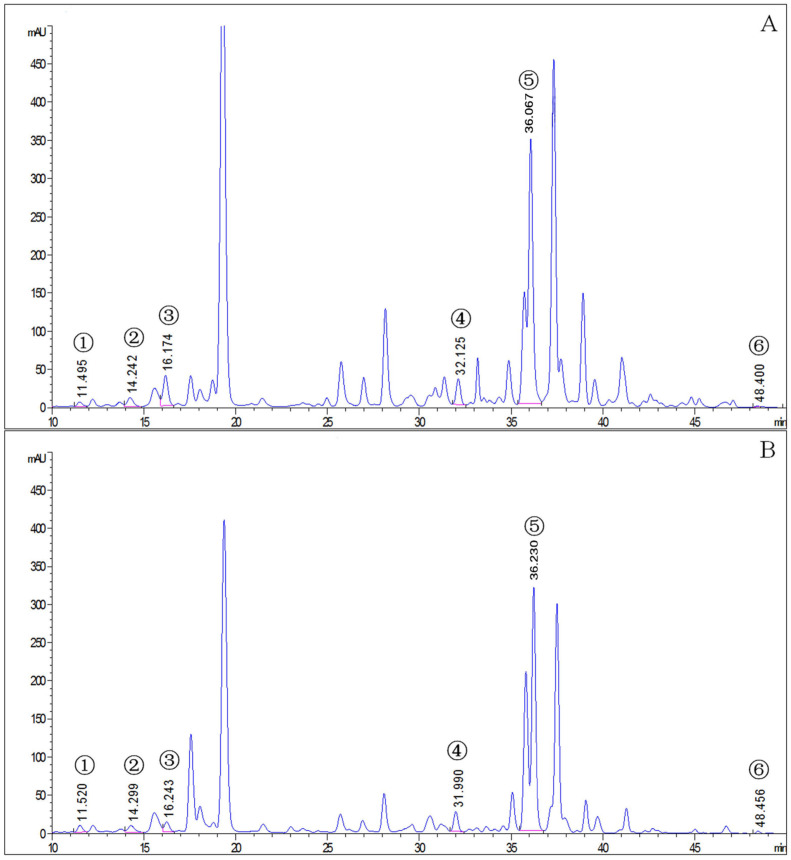
The chromatogram of carotenoids in *T. cinnabarinus* and *T. urticae*. (**A**), *T. cinnabarinus*. (**B**), *T. urticae*. 1, neoxanthin; 2, cucurbitaxanthin A; 3, astaxanthin; 4, α-carotene; 5, β-carotene; 6, γ-carotene.

**Figure 2:**
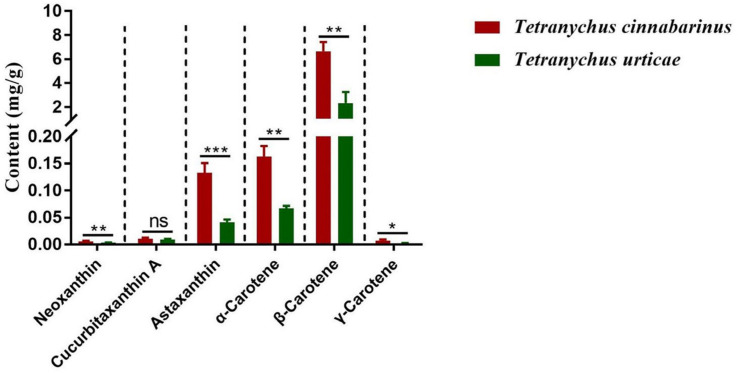
The contents of carotenoids in *T. cinnabarinus* and *T. urticae*. Note: The bar graph represents mean ± SE; The dotted line is to distinguish between different carotenoids; Asterisks on the error bars show significant differences between *T. cinnabarinus* and *T. urticae* (* *p* < 0.05) (** *p* < 0.01) (*** *p* < 0.001) (ns, no significance).

The mite carotenoid profiles in Figure 1 display that the *T. cinnabarinus* and *T. urticae* chromatograms overlapped with no difference in carotenoid types, and all six carotenoid standards were found in *T. cinnabarinus* and *T. urticae* (Figure S1). The separation of the six carotenoids was good, corresponding to the peak times and peak shapes of the standards one by one. The structural formulae of the six carotenoid molecules are extremely similar, as they are all derived from the C_40_ backbone with various modifications. The molecular formulae of the three non-oxygenated carotenoids are the same, and the differences in the molecular formulae are due to changes in the positions of the carbon–carbon double bonds. The quantitative analysis revealed that *T. cinnabarinus* has a higher total carotenoid content and more individual carotenoids except for cucurbitaxanthin A than *T. urticae* (Figure 2), with β-carotene being the most abundant carotenoid in both mites.

**Figure 3:**
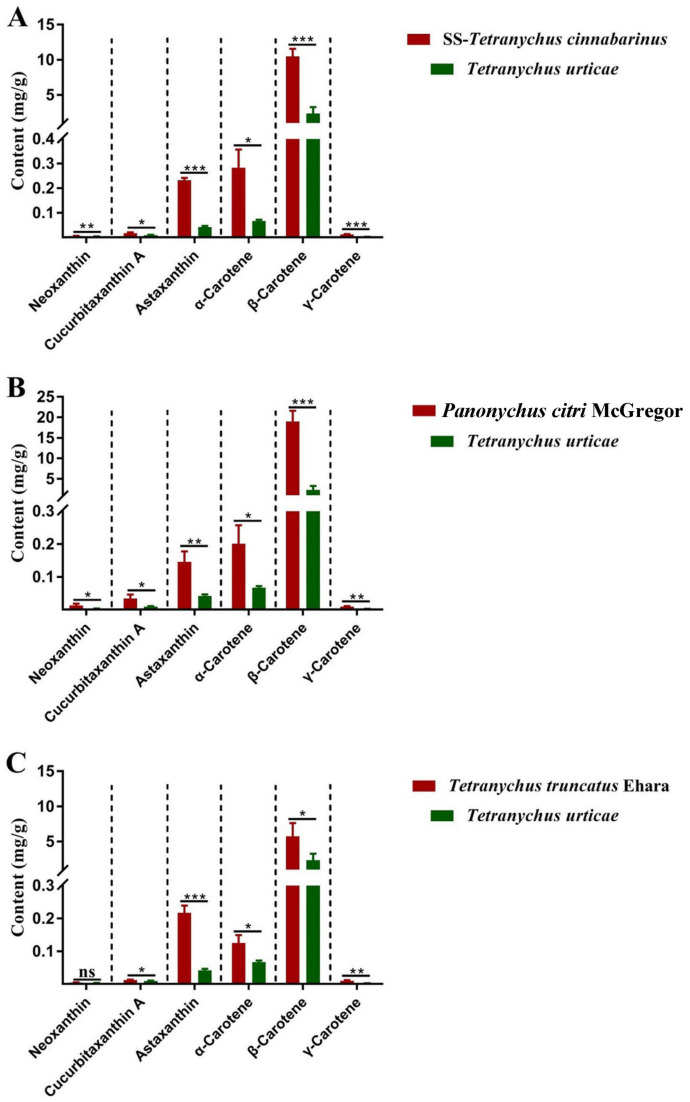
The contents of carotenoids in red-type mites and *T. urticae*. (**A**) the contents of carotenoids in SS-*T. cinnabarinus* and *T. urticae*; (**B**) the contents of carotenoids in *Panonychus citri* McGregor and *T. urticae*; (**C**) the contents of carotenoids in *Tetranychus truncatus* Ehara and *T. urticae*. Note: Bar graph represents mean ± SE; The dotted line is to distinguish between different carotenoids; Asterisks on the error bars display significant differences between red spider mites and *T. urticae* (* *p* < 0.05) (** *p* < 0.01) (*** *p* < 0.001) (ns, no significance).

These results were then verified in three other red-type mite populations, the indoor reared *T. cinnabarinus* strain (SS), *P. citri*, and *T. truncates* (Figure 3). There was a greater carotenoid content in the three red mites than in *T. urticae*, and β-carotene was the most abundant carotenoid in all mites. All six carotenoids were higher in SS-*T. cinnabarinus* and *P. citri* than in *T. urticae*. Besides neoxanthin, *T. truncates* exhibited higher levels of the other five carotenoids than *T. urticae*.

### 3.2. Transcriptome Sequencing and Carotenoid-Related DEGs Analysis

**Figure 4:**
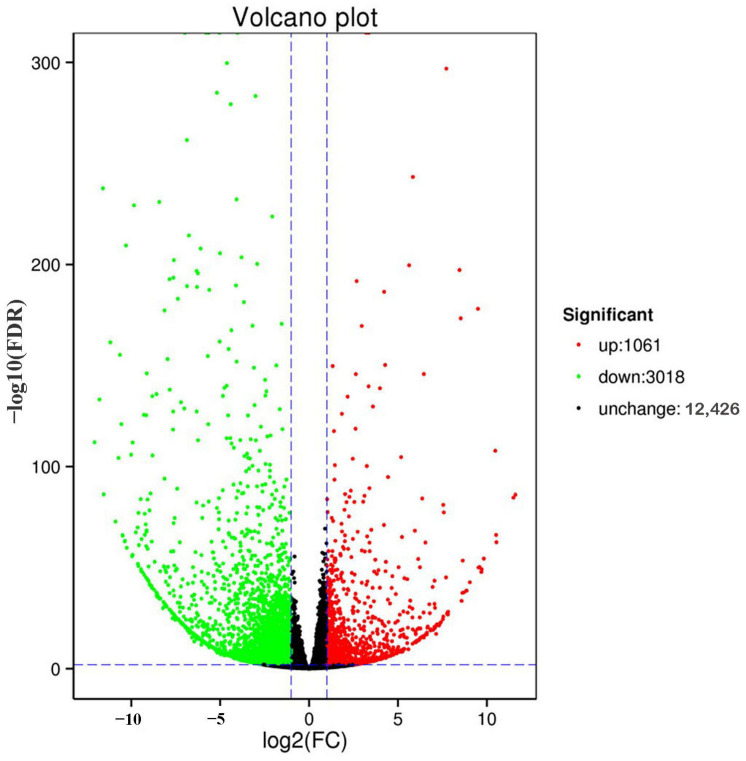
Volcano plot of gene expression between *T. cinnabarinus* and *T. urticae*. Note: The vertical dotted line on the right indicates that the expression factor is up-regulated by two times, the vertical dotted line on the left indicates that the expression factor is down-regulated by two times, and the horizontal dotted line indicates that the significance *p*-value is 0.05.

Transcriptome sequencing of the six mite samples obtained 99.38 GB of clean data, with 94.02% Q30. The transcriptome data analysis revealed 4079 DEGs in *T. cinnabarinus* and *T. urticae*, of which 1061 were upregulated in *T. cinnabarinus,* and 3018 were upregulated in *T. urticae* (Figure 4).

**Figure 5:**
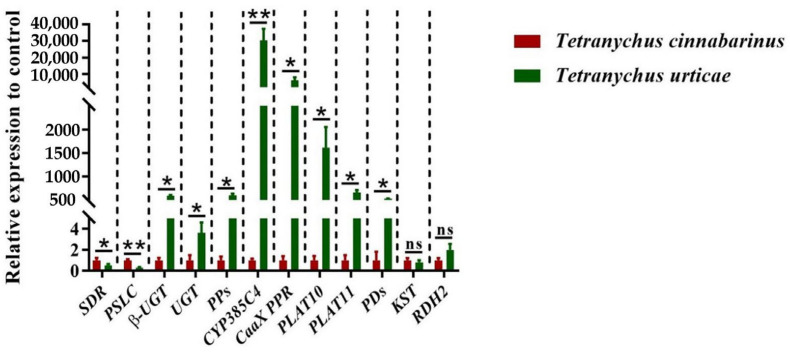
Expression levels of 12 genes in *T. cinnabarinus* and *T. urticae*. The bar graph represents mean ± SE; Asterisks on the error bars show significant differences between *T. cinnabarinus* and *T. urticae* (* *p* < 0.05) (** *p* < 0.01) (ns, no significance); the expression level of *T. cinnabarinus* was set as 1.

According to functional annotation, 12 DEGs related to carotenoid metabolism synthesis were identified (Table 1). The quantitative primers designed for the 12 genes were amplified by standard curves, and the amplification efficiencies were all in the range of 90% to 110%, which can be used for subsequent qPCR. The expression of these 12 DEGs was quantified by qPCR, showing that the expression was significantly different between the two mites, except for two genes: *KST* and *RDH2* (Figure 5). Two genes, *SDR* and *PSLC*, were highly expressed in *T. cinnabarinus*, whereas *β-UGT*, *UGT*, *PPs*, *CYP385C4*, *CaaX PPR*, *PLAT10*, *PLAT11*, and *PDs* were highly expressed in *T. urticae*. The differential expression patterns of these ten genes were consistent with the transcriptome results.

### 3.3. Analysis of Differential Gene Expression Patterns

**Figure 6:**
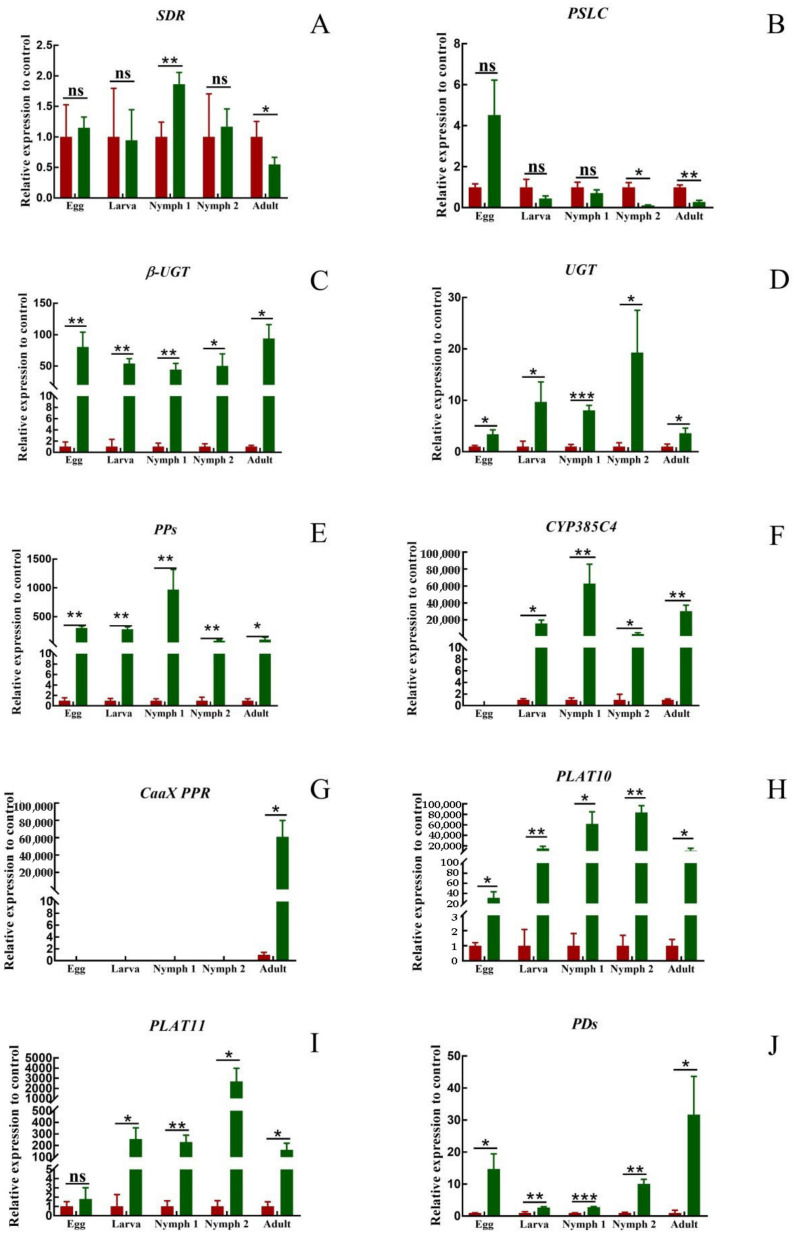
(**A**–**J**) Expression pattern of ten genes in *T. cinnabarinus* and *T. urticae.* The bar graph represents mean ± SE; Asterisks on the error bars show significant differences between *T. cinnabarinus* and *T. urticae*, the green bars represent *T.urticae*, the red bars represent *T.cinnabarinus* (* *p* < 0.05) (** *p* < 0.01) (*** *p* < 0.001) (ns, no significance); the expression level of *T. cinnabarinus* was set as 1.

There was no significant pattern in the expression of *SDR* and *PSLC* at different developmental stages (Figure 6A,B). At the egg and larva stages, there was no significant difference in the expression of the two genes in *T. cinnabarinus* and *T. urticae*. At the nymph I stage, the expression of *SDR* in *T. cinnabarinus* was lower than that in *T. urticae*, and the expression of *PSLC* was still not different. At the nymph II stage, there was no difference in the expression of *SDR*, and the expression of *PSLC* was higher in *T. cinnabarinus* than in *T. urticae*. At the adult mite stage, the expression of both genes was higher in *T. cinnabarinus* than in *T. urticae*. The eight genes that were highly expressed in adult mites of *T. urticae* were more highly expressed than those of *T. cinnabarinus* in all stages (Figure 6C–J). However, the expression of *CaaX PPR* was not detected in eggs, larva, or the nymph I and II stages. Only in the adult mite stage, the expression of the *T. urticae* was 6115.17 times higher than that of *T. cinnabarinus* (Figure 6G).

### 3.4. Functional Validation of Carotenoid-Related DEGs

**Figure 7:**
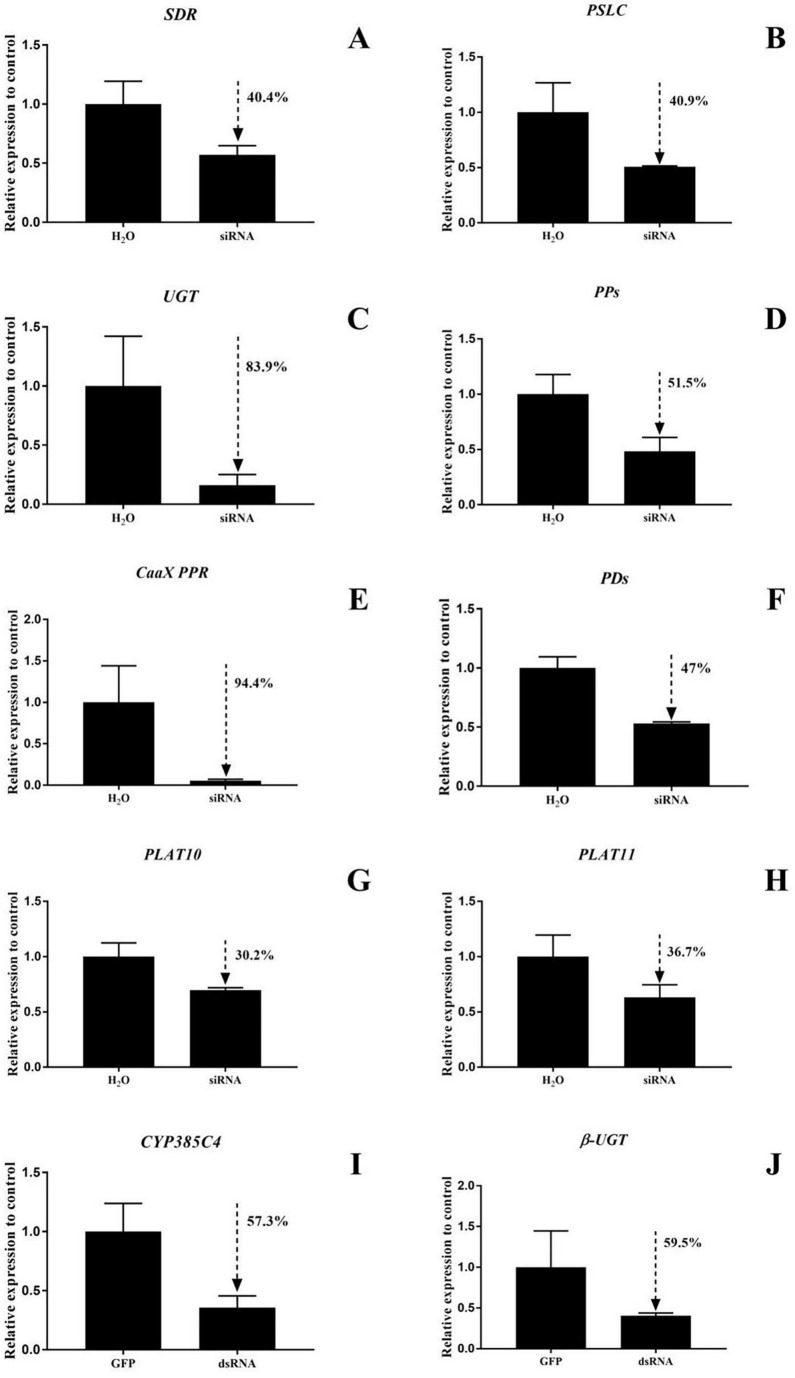
(**A**–**J**) Detection of 10 gene silencing efficiencies in mites. The bar graph represents mean ± SE; the dashed arrows and the percentage next to them indicate the efficiency of RNAi; the expression level of control (GFP and H_2_O) was set as 1.

RNAi function validation was performed on the ten DEGs, and the siRNA silencing efficiencies of *SDR* and *PSLC* in *T. cinnabarinus* were 40.4% and 40.0%, respectively. In *T. urticae*, the siRNA silencing efficiencies of *UGT*, *PPs*, *CaaX PPR*, *PDs*, *PLAT10*, and *PLAT11* were 83.9%, 51.5%, 94.4%, 47%, 30.2%, and 36.7%, with dsRNA silencing efficiencies of *CYP385C4* and *β-UGT* of 57.3% and 59.5%, respectively (Figure 7).

**Figure 8:**
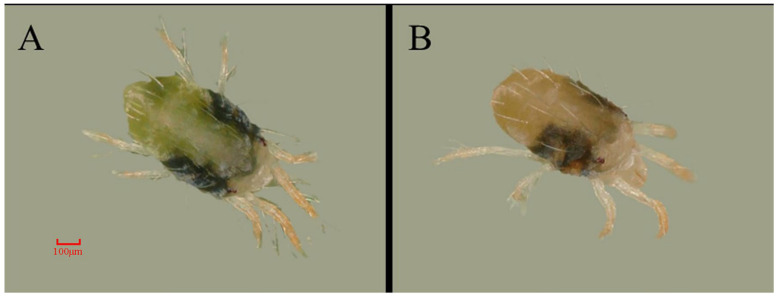
The phenotype of *T. urticae* after the silencing of *β-UGT* and *UGT*. (**A**): GFP control. (**B**): A mixture of *β-UGT*-dsRNA and *UGT*-siRNA.

HPLC analysis revealed that silencing seven genes affected the carotenoid content in mites (Table 2). After silencing the *SDR*, the amount of six carotenoids in *T. cinnabarinus* did not alter significantly. The γ-carotene content in *T. cinnabarinus* was reduced by 20% after *PSLC* silencing. In *T. urticae*, there was no significant change in the content of six carotenoids after silencing *PPs* and *CaaX PPR*, but silencing *CYP385C4*, *PLATs*, and *PDs* increased β-carotene content to 20%, 64%, and 41%, and γ-carotene increased to 29%, 98%, and 142%, respectively. After silencing *β-UGT* and *UGT*, α-carotene increased by 41% and 132%, β-carotene increased by 48% and 59%, and γ-carotene increased by 39% and 91% in *T. urticae*. Furthermore, the body color of *T. urticae* became significantly yellow after being fed with a mixture of *β-UGT*-dsRNA and *UGT*-siRNA for two days, as illustrated in Figure 8, which was consistent with the carotenoid accumulation.

## 4. Discussion

The taxonomic status of the *T. cinnabarinus* and *T. urticae* subspecies had been controversial, and evidence from differences in the epidermal coloration as to whether they belong to the same species is crucial. In the present study, the use of *T. cinnabarinus* and *T. urticae* harvested from the same area and the same host was analyzed to avoid the influence of other factors on the results. Polymorphism in aphid body color was caused by differences in the type and content of carotenoids, which are also the most important pigment components of mite epidermis [18,25]. The total carotenoid content of the three red-type mites, *T. cinnabarinus*, *P. citri*, and *T. truncatus*, was higher than that of *T. urticae*, probably due to increased carotenoid metabolism in *T. urticae*, implying that the difference in carotenoid content was responsible for color difference. In *T. urticae*, β-carotene acts as a precursor to perceive the photoperiod induction, thus inducing diapause, and the carotenoid metabolic pathway is completely dependent on endogenously produced β-carotene [7,10,26]. A recent study demonstrated that the variation of the yellow and green body color of locusts depends on changes in β-carotene content [7]. β-carotene, as the most abundant carotenoid mites, may play an important role in regulating body color.

The transcriptome analysis of *T. urticae* (red and green types) from five different geographical populations confirmed that pigment-related DEGs were mainly distributed in four synthetic pathways: heme, melanin, ophthalmic pigment, and retinoid [20]. Carotenoids are the main pigments in *T. urticae,* but the metabolic pathways have not been determined [27,28,29]. Transcriptome sequencing studies can help reveal how pigment-related genes regulate body color and identify 12 DEGs related to carotenoid metabolism. According to qPCR validation, these DEGs were consistent with the transcriptome, except for *KST* and *RDH2. PSLC* is mainly associated with carotenoid synthesis, and the high expression of *PSLC* may have promoted the synthesis of more carotenoids in *T. cinnabarinus* than in *T. urticae*. Seven DEGs (*SDR*, *UGT*, *PPs*, *PDs*, *β-UGT*, *CYP385C4*, and *CaaX PPR*) are associated with the downstream metabolism of β-carotene, and except for *SDR*, the expression of these genes was lower in *T. cinnabarinus* than in *T. urticae*, which is consistent with the lower β-carotene content in *T. urticae*. It has been reported that *Candida antarctica* lipase enzymatically carotenoid ester to obtain free neoxanthin, and two lipase genes (*PLAT10*, *PLAT11*) may play a similar role in mites [30]. Thus, these DEGs may play an important role in the carotenoid pathway of mites, and are directly or indirectly involved in the mechanism that contributes to the formation of the body color differences between *T. cinnabarinus* and *T. urticae*.

The 10 carotenoid-related DEGs showed an excellent full-length sequence similarity in the two mites, suggesting that their physiological functions may not differ. In addition, the results of expression pattern analysis showed that the expression of these genes varied greatly at different developmental stages, suggesting that the differences in gene expression may be the main cause of the red-green morphology. The increased expression of the gene *PSLC* in the *T. cinnabarinus* nymph II stage may be related to the deepening of the body color. Some studies had found that the carotenoid synthesis gene (*PSLC*) was differentially expressed in the non-diapause and diapause female adult mites, and differences in gene expression may lead to genotypic variation in genetically similar species [31,32,33]. It has been demonstrated that pigment-related genes can affect body color in different species, and differential expression or the deletion of genes can cause polymorphism in the phenotypes of the same species, but there are fewer studies related to the mechanism of regulating body color differences in mites [17,34,35,36,37,38,39]. A carotenoid desaturase gene, tetur01g11270, was involved in the process of pigment synthesis in *T. urticae*, but it is not clear whether other related genes also play a role in this process in other different populations [10].

The differential carotenoid-related genes were functionally validated by RNAi and HPLC techniques, and the silencing of seven genes caused changes in the carotenoid profiles of *T. cinnabarinus* or *T. urticae*. PSLC encodes carotenoid synthesis/cyclase, and it has been reported that the injection method RNAi did not cause phenotypic changes in T. urticae [40]. It is known that SDR is involved in the transport of carotenoids and plays a role in the accumulation of carotenoids in the cells [41]. In this study, silencing *PSLC* resulted in only a 20% reduction of γ-carotene in *T. cinnabarinus*, suggesting that this gene may not play a key role in the formation of body color. The silencing of *CYP385C4* did not cause changes in the content of oxygenated carotenoids in *T. urticae*, suggesting that *CYP385C4* may not be highly relevant to the convergent evolution of ketocarotenoids in mites. The silencing of six DEGs of *T. urticae* affected the level of only three hydrocarbon carotenoids: α-carotene, β-carotene, and γ-carotene. The weak pigmentation changes in *T. urticae* after *β-UGT* and *UGT* silencing suggested that the formation of the red-green phenotype is a complex multifactorial mechanism. The formation of the differences in body color between *T. cinnabarinus* and *T. urticae* may also be related to the different proportions of multiple carotenoid complexes. These results suggest that the genes *PDs*, *PLAT10*, *PLAT11*, and *CYP385C4* are involved in carotenoid anabolism in mites, but the specific pathways need to be further investigated.

There were no differences in the carotenoid types between the red-green mites; only the total content varied, and seven genes were identified as responsible for this variation. It is clear from the present results that mite carotenoid pathways are more complex than previously thought, so they must be studied systematically and thoroughly. The carotenoid-related genes screened in this study may only be an indirect cause of the differences in body color between *T. cinnabarinus* and *T. urticae*. A combination of genes and the environment may be the direct cause of the differences in body color between the two species. The increased pigment metabolism in *T. urticae* compared to *T. cinnabarinus* may allow it to better adapt to different environments. The RNAi technique has some limitations, so future studies using CRISPR/Cas9 may provide more definitive results [10]. In conclusion, this study established a method for mite pigment analysis and provided a basis for refining pigment metabolism pathways in mites.

## Figures and Tables

**Table 1:** The list of DEGs related to carotenoid pathway.

T. urticae ID	Abbreviation	Full Name
tetur17g01350	*KST*	Ketoacyl-synthetase, C-terminal extension
tetur05g02630	*SDR*	Short-chain dehydrogenase/reductase
tetur21g01400	*UGT*	UDP-glycosyltransferase
tetur02g13630	*PPs*	Protein prenyltransferase
tetur01g11260	*PSLC*	lycopene cyclase/phytoene synthase
tetur11g04810	*PDs*	phytoene desaturase
tetur08g00550	*RDH2*	Putative retinol dehydrogenase
tetur05g05060	*β-UGT*	Sterol 3-beta-glucosyltransferase
tetur11g05720	*PLAT10*	Lipase/lipooxygenase; PLAT/LH2
tetur11g05520	*CYP385C4*	Cytochrome P450
tetur11g05730	*PLAT11*	Lipase/lipooxygenase; PLAT/LH2
tetur13g01900	*CaaX PPR*	CaaX prenyl protease Rce1

**Table 2:** Change of carotenoids after genes were silenced.

Gene	(TU/TC) Fold Change	Mite	RNAi Efficiency	Change of Carotenoids		
				α-Carotene	β-Carotene	γ-Carotene
*SDR*	0.55	TC	40.4%	-	-	-
*PPs*	101.72	TU	51.5%	-	-	-
*CaaX PPR*	6115.17	TU	94.4%	-	-	-
*PSLC*	0.28	TC	40.9%	-	-	−20%
*CYP385C4*	30,323.34	TU	57.3%	-	+20%	+29%
*PDs*	31.70	TU	47.0%	-	+41%	+142%
*PLAT10*	1114.28	TU	30.2%	-	+64%	+98%
*PLAT11*	164.60	TU	36.7%	-	+64%	+98%
*β-UGT*	93.71	TU	59.5%	+41%	+48%	+39%
*UGT*	3.62	TU	83.9%	+132%	+59%	+91%

TC: *T. cinnabarinus*; TU: *T. urticae*; “-” means no significant difference.

## Data Availability

The data presented in this study are available in the article.

## Supplementary Materials

The following supporting information can be downloaded at: https://www.mdpi.com/article/10.3390/insects14100823/s1, Table S1: Primer used for qPCR analysis; Table S2: Specific primers used for RNAi; Figure S1: The chromatogram of six carotenoid standards.
