# Supplementary material for: Uridine Diphosphate Glycosyltransferases (UGTs) Involved in the Carotenoid-Based Body Color Difference between Tetranychus cinnabarinus (Red) and Tetranychus urticae (Green)
Source: Insects. 2023 Oct 18;14(10):823. doi: 10.3390/insects14100823 (PMC10607543; doi:10.3390/insects14100823)
Supplement: Supplementary file 1 [file insects-14-00823-s001.zip › insects-2556148-supplementary.pdf]

Table S1. Primer used for qPCR analysis.

Gene	Sequences (5´ to 3´)	Length (bp)
KST	RT-F: TATTGTGCACGGATTTTGGA<br>RT-R: CCATGAGAGCGCTTGTTGTA	186
SDR	RT-F: TGGATCAACAAGGCTTTCCTA<br>RT-R: GCCAGCAGTTTTCAGTTCAAG	174
UGT	RT-F: CCAAACATGTGGGGTGAAA<br>RT-R: GATTCGAGCACCAATTCCA	195
PPs	RT-F: TCAAGTTTGGCAGCATCG<br>RT-R: CCATCCCACCATTGGAAA	156
PSLC	RT-F: GCAGCATTCCGTTCACTGAC<br>RT-R: TCCCATGCATAGCCTCTGGA	83
PDs	RT-F: TAAGGATATGGACGCACTGACC<br>RT-R: TCAACCTTATCATCGAAACCACCT	81
RDH2	RT-F: CATTTTGCCCTGGTCCTG<br>RT-R: CCCGGTGACTGAATCGAG	167
β-UGT	RT-F: AAAGTGGTTCAAAACGTGGATT<br>RT-R: TTGACACGAAAACCAATCTTCTT	161
PLAT10	RT-F: GAAGGGCATAGACTGCAGAA<br>RT-R: GTTCTACCATGGGCCCACAA	100
CYP385C4	RT-F: GGACCAGAACCTCCTTCCA<br>RT-R: TGACGCAAACAGCTCGAA	187
PLAT11	RT-F: GAATTGCACATGCCGATG<br>RT-R: TATTACCATGGGCCCACAAG	53
CaaX PPR	RT-F: GGTGATTCCCCTTGGGATA<br>RT-R: TGGCTGAGCGAGAAAAGAA	240
RPS18	RT-F: ACGTGCTGGTGAACTTACCGAAGA<br>RT-R: TGCCTATTCAAGAACCAAAGTGGG	99

Note: RT-F: Forward primer; RT-R: Reverse primer.

Table S2. Specific primers of used for RNAi.

Gene	Sequences (5´ to 3´)
SDR	Oligo1: GGATCCTAATACGACTCACTATAGAAGCTTTACGAACTGACC
	Oligo2: AAGGTCAGTTCGTAAAGCTTCTATAGTGAGTCGTATTAGGATCC
	Oligo3: GGATCCTAATACGACTCACTATAGGTCAGTTCGTAAAGCTTC
	Oligo4: AAGAAGCTTTACGAACTGACCTATAGTGAGTCGTATTAGGATCC
UGT	Oligo1: GGATCCTAATACGACTCACTATAGAAATGTCCTCATCGTTTC
	Oligo2: AAGAAACGATGAGGACATTTCTATAGTGAGTCGTATTAGGATCC
	Oligo3: GGATCCTAATACGACTCACTATAGAAACGATGAGGACATTTC
	Oligo4: AAGAAATGTCCTCATCGTTTCTATAGTGAGTCGTATTAGGATCC
PPs	Oligo1: GGATCCTAATACGACTCACTATAGTCCCAGAAAATGAAAGTC
	Oligo2: AAGACTTTCATTTTCTGGGACTATAGTGAGTCGTATTAGGATCC
	Oligo3: GGATCCTAATACGACTCACTATAGACTTTCATTTTCTGGGAC
	Oligo4: AAGTCCCAGAAAATGAAAGTCTATAGTGAGTCGTATTAGGATCC
PSLC	Oligo1: GGATCCTAATACGACTCACTATAGCCAGTGAACCAGATTTAC
	Oligo2: AAGTAAATCTGGTTCACTGGCTATAGTGAGTCGTATTAGGATCC
	Oligo3: GGATCCTAATACGACTCACTATAGTAAATCTGGTTCACTGGC
	Oligo4: AAGCCAGTGAACCAGATTTACTATAGTGAGTCGTATTAGGATCC
PDs	Oligo1: GGATCCTAATACGACTCACTATAGGTGATGCTAAGGAAAAAC
	Oligo2: AAGTTTTTCCTTAGCATCACCTATAGTGAGTCGTATTAGGATCC
	Oligo3: GGATCCTAATACGACTCACTATAGTTTTTCCTTAGCATCACC
	Oligo4: AAGGTGATGCTAAGGAAAAACTATAGTGAGTCGTATTAGGATCC
PLAT	Oligo1: GGATCCTAATACGACTCACTATAGAATTGGTGAAGAGACAAC
	Oligo2: AAGTTGTCTCTTCACCAATTCTATAGTGAGTCGTATTAGGATCC
	Oligo3: GGATCCTAATACGACTCACTATAGTTGTCTCTTCACCAATTC
	Oligo4:

CaaX PPR	AAGAATTGGTGAAGAGACAACTATAGTGAGTCGTATTAGGATCC<br>Oligo1: GGATCCTAATACGACTCACTATAGGTATGTTCATGTCAGCAC<br>Oligo2:<br>AAGTGCTGACATGAACATACCTATAGTGAGTCGTATTAGGATCC<br>Oligo3:<br>GGATCCTAATACGACTCACTATAGTGCTGACATGAACATACC<br>Oligo4:<br>AAGGTATGTTCATGTCAGCACTATAGTGAGTCGTATTAGGATCC
CYP385C4	RNAi-F: TAATACGACTCACTATAGGGGGCTTCTTCGAGTGAACCAG<br>RNAi-R: TAATACGACTCACTATAGGGACAGGGTTGATGTCGTTTCC
β-UGT	RNAi-F: TAATACGACTCACTATAGGGCTATGATTTGGATGAAGA<br>RNAi-R: TAATACGACTCACTATAGGGTCAAGATGAACTTTTTTG

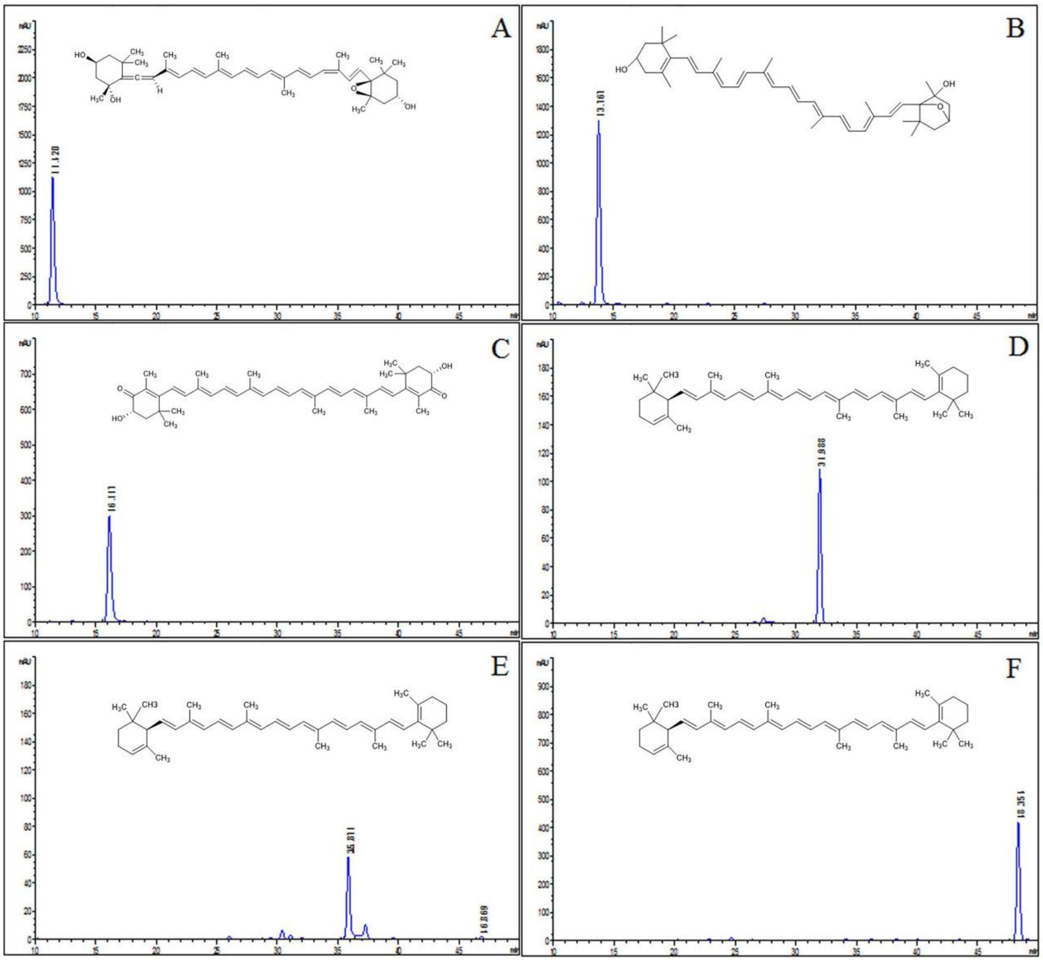

Figure S1. The chromatogram of 6 carotenoid standards.

A, neoxanthin; B, cucurbitaxanthin A; C, astaxanthin; D, α-carotene; E, β-carotene; F, γ-carotene.
